# Antipsychotic polypharmacy and associated factors among patients with schizophrenia: Multicenter cross-sectional study in Northwest Ethiopia

**DOI:** 10.1371/journal.pone.0290037

**Published:** 2023-08-14

**Authors:** Fasil Bayafers Tamene, Faisel Dula Sema, Ashenafi Kibret Sendekie

**Affiliations:** 1 Clinical Pharmacy Unit, Pharmacy Department, Health Science College, Debre Markos University, Debre Markos, Ethiopia; 2 Department of Clinical Pharmacy, School of Pharmacy, College of Medicine and Health Sciences, University of Gondar, Gondar, Ethiopia; Dilla University College of Health Sciences, ETHIOPIA

## Abstract

**Background:**

Antipsychotic polypharmacy (APP) remains common despite guideline recommendations to minimize combinations, except after repeated antipsychotic monotherapy trials. This study aimed to assess APP and its associated factors among schizophrenia patients at comprehensive specialized hospitals in Northwest Ethiopia.

**Methods:**

An institutional-based cross-sectional study was conducted among 422 schizophrenia patients at selected hospitals in Ethiopia from June to August 2022. The data were collected using a semi-structured questionnaire. Study participants were enrolled using systematic random sampling. Data entry and analysis were done with Epi-data version 4.6.1 and SPSS version 24, respectively. APP was determined by reviewing the number of medications based on relevant evidence. A multivariable logistic regression model was fitted to identify APP factors. Variables with a p-value of < 0.05 at a 95% confidence interval were considered statistically significant.

**Results:**

From a total of 430 approached samples, 422 (98.1% response rate) eligible patients were included in the study. An overall APP prevalence was 22.7% (95% CI: 19–27). Duration of illness (AOR = 2.88; 95% CI: 1.49, 5.59); duration of treatment (AOR = 3.79; 95% CI: 1.05, 13.62); number of admissions (AOR = 4.93; 95% CI: 2.52, 9.64); and substance use (AOR = 2.58; 95% CI: 1.49, 4.47) were significantly associated with APP.

**Conclusion and recommendation:**

In this study, APP was recorded in a considerable number of patients. Patients with a longer duration of illness and treatment, frequent admissions, and substance users need critical follow-up to minimize antipsychotic medication use.

## Introduction

Antipsychotic polypharmacy refers to the co-prescription of more than one antipsychotic drug for a patient. In the treatment of schizophrenia or other psychotic diseases, antipsychotic polypharmacy (APP) is frequently used [1, 2]. According to a comprehensive meta-analysis, the worldwide prevalence of APP is 19.6%, with the most prevalent combinations comprised of first-generation antipsychotics (FGAs) and second-generation antipsychotics (SGAs) [3]. In Africa, a systematic review and meta-analysis found that the incidence of APP in schizophrenic patients was 40.6%. The most frequently prescribed antipsychotic combinations were depot and oral FGA [4]. In Ethiopia, a hospital-based study undertaken on schizophrenia patients with follow-up concluded that APP magnitude was high (28.2%) [2].

A typical rationale for prescribing more than one antipsychotic is to get a better or faster therapeutic response than antipsychotic monotherapy. However, treatment recommendations and supporting data for APP’s safety and effectiveness are inadequate [2]. Problems related to the prescription of combined antipsychotics have been clinically linked with several drawbacks, such as increased anticholinergic demand, shorter observation duration, greater sickness intensity, lower antidepressant usage, frequent admissions, and prolonged therapy duration [2, 3]. In addition, APP is associated with the increased incidence and severity of adverse drug reactions. Besides this, APP is associated with reduced health-related quality of life [5], reduced adherence, a higher risk of medication interactions [6], a substantial financial burden on patients and the public health system [7], and more medication errors due to treatment complexity. Furthermore, there is some data showing that APP may be related to cognition impairments; however, this appears to be largely a function of total daily dosage rather than the number of antipsychotics administered [8].

Although there are a significant number of schizophrenia patients receiving antipsychotic medication at comprehensive specialized hospitals in Northwestern Ethiopia, no published studies are available that assess the magnitude of APP specific to the study area. Furthermore, this study assessed the physical comorbidity of the patients; substance use was evaluated using a standard tool; and the medication were briefly indicated. It also identified those medications considered APP that were not addressed in previous studies. More importantly, this study was conducted at a multicenter, which gives the opportunity for generalization. In addition, the study will add to the body of knowledge for patients, caregivers, and healthcare providers in the area regarding APP and potential variables associated with APP in patients with schizophrenia. Therefore, this study aimed to assess APP and its associated factors among patients with schizophrenia at comprehensive specialized hospitals in Northwest Ethiopia.

## Materials and methods

### Study setting, period and design

This study was conducted at the University of Gondar Comprehensive Specialized Hospital (UoGCSH), Felege-Hiwot Comprehensive Specialized Hospital (FHCSH), and Tibebe-Ghion Comprehensive Specialized Hospital (TGCSH). UoGCSH is located in Gondar, a city 730 kilometers Northwest of Addis Ababa (the capital city of Ethiopia). UoGCSH serves 12,000 patients with psychiatric disorders annually. The psychiatry unit of UoGCSH contains two beds in the emergency room, nineteen beds for inpatient care, and four outpatient departments. FHCSH and TGCSH are located in Bahir Dar, 521 kilometers away from Addis Ababa. Felege-Hiwot comprehensive specialized hospital serves for 19,200 patients with psychiatric disorders annually and has seven inpatient beds and six outpatient sections. Tibebe-Ghion comprehensive specialized hospital has one emergency room and two inpatient rooms with six beds. The study was conducted cross-sectionally from June 1 to August 30, 2022.

### Population inclusion and exclusion criteria

Adult patients with schizophrenia in outpatient departments at the selected comprehensive specialized hospitals in northwest Ethiopia used as a source population. All adult patients with schizophrenia having regular follow-ups in the outpatient departments of UoGCSH, FHCSH, and TGCSH during the study period were included in the study populations. Patients 18 years and above, taking antipsychotic medication, who had insight to respond to oral questions (satisfied the requirement in the insight assessment tool (got 3 out of 3)), and patients who had one or more previous visits, were included in the study. Patients with incomplete medical records were excluded from the study.

### Sample size determination

The sample size for the application was calculated using a single population proportion formula as follows: *n* = *Z* 2 * *P* (1 –*p*)/*w*2

Where n is the desired sample size for a population of >10,000, Z is the typical normal distribution set at 1.96 (which corresponds to 95% CI), the p-value signifies that positive prevalence was utilized in calculating the optimal sample size, and W is the degree of accuracy required (a marginal error is 0.05). In the previous study done on APP prevalence, it was estimated to be 28.2% [2]. Then, computing for n = 1.96^2^*0.282(1–0.282)/0.05^2^; n = 308; by adding 10% non-respondents, the estimated sample size was 339. However, for the factors associated with APP, taking AOR from previous literature [2], it was calculated as duration of treatment, 5–10 years; outcome exposed to unexposed (18.68%); calculating with AOR yielded 429.5. For the duration of treatment > 10 years, the outcome exposed to unexposed (18.68%); computing with AOR gave 284. Finally, for the number of admissions, the outcome exposed to unexposed (22.41%); computing with AOR, gave 126. Consequently, from these varaibles’ sample size calculations, the largest calculated (429.5 ≈ 430) sample size was used.

### Sampling technique and procedure

Comprehensive specialized hospitals in northwestern Ethiopia were selected randomly using lottery method. The total number of schizophrenia patients on follow-up within 3 months was taken from the patients’ registration documents to allocate samples proportionally within study areas. After proportional allocation, a systematic random sampling technique was used to select study participants. The sampling fraction (k) was calculated by dividing the total number of schizophrenic patients within 3 months in the study area by the overall sample size (2625/430 gives 6.1 ≈ 6). The average number of patients within 3 months at UoGCSH, FHCSH, and TGCSH was 1100, 1150, and 375, respectively. So, the proportional allocation of sample size was 180 for UoGCSH, 182 for FHCSH, and 62 for TGCSH. The starting point was chosen from 1 to 6. Participants were interviewed, and relevant data were reviewed from medical charts for every sixth patient. This was one until the sample requirement was fulfilled. A unique patient identification card number was used as a questionnaire code to prevent the same patient from participating in the study more than once.

### Study variables

Antipsychotic polypharmacy was the dependent variable. The independent variables were the sociodemographic characteristics of the participants (like marital status, residence, educational status, occupation, and monthly income), and patient-related conditions including the number of admissions, presence of comorbidity, duration of illness, duration of treatment, and substance use.

### Operational definitions

**Antipsychotic polypharmacy:** refers to the co-prescription of more than one antipsychotic drug for a particular patient for at least one month and above [2, 9]. Polypharmacy was determined by reviewing the number of medications in the last follow-up medication record.

**Substance use** (current users): refers to using at least one specific substance (alcohol, khat, or cigarettes) for nonmedical purposes within the last 3 months, according to the Alcohol, Smoking, and Substance Involvement Screening Tool (ASSIST) [10].

### Data collection instrument, procedures, quality control

A semi-structured questionnaire was adopted from previous literature [2] with some modifications for the context of the study area and the socio-demographic characteristics of study participants. It was translated into the local Amharic language and then back-translated to the English version to check consistency. The translation was not required for variables obtained from patients’ medical records. The data collected by the patient interview includes sociodemographic characteristics and substance use. The patients’ medical charts were used to fill in clinically related variables like duration of illness, duration of treatment, number of admissions, presence of comorbidity, type of antipsychotic, and presence of antipsychotic polypharmacy. The data collection tool included three parts. The first part contained the socio-demographic characteristics of the study participants, such as sex, age, marital status, residence, religion, educational level, occupation, and income level. The second section consisted of clinical and medication-related characteristics like duration of illness, presence of comorbidity, the patient’s medication record, duration of treatment, and number of admissions. The third section consisted of the current substance use assessment tool. ASSIST was designed for a briefly screening patients’ use of psychoactive substances, and it was developed and validated by the WHO [10].

The data were collected by five bachelor psychiatrists in a face-to-face interview using a pretested questionnaire and supervised by senior psychiatrists and clinical pharmacists in the respective hospitals. The supervisors distributed all the necessary items to data collectors on each data collection. They also checked the data collection questionnaire for completeness during the data collection period.

To assure the consistency and quality of the data, one day of training was given to the data collectors in each study area. A pretest was conducted on 5% of schizophrenia patients (around 22 patients) at Dessie Comprehensive Specialized Hospital’s outpatient department to identify potential problems with the data collection tool and check the consistency of the questionnaire. Some modifications, such as the correction of typing errors and the rearranging of questionnaires, were made. The ASSIST’s internal consistency was assessed and found in the acceptable range with a Cronbach’s Alpha of 0.76.

### Data entry and analysis

The collected data was cleaned, coded, and entered into Epi Data 4.6.0 and analyzed using Statistical Package for Social Studies (SPSS) version 24. In the descriptive analysis, the mean with standard deviation (SD), frequency, and percentages were used to check the distribution of data. Univariable and multivariable binary logistic regression analyses were employed to identify APP factors. The model’s fitness was tested, and the Hosmer and Lemeshow test result was 0.797. Multicollinearity was checked, and the maximum variation inflation factor (VIF) reported was less than 5, which was within the acceptable level. Variables with a p-value < 0.2 in the univariable analysis were further analyzed in the multivariable analysis. The odds ratio (OR) with a 95% confidence interval was computed for each variable along with the corresponding p-value to see the strength of the association. A P-value of < 0.05 was used as the cut-off for the significance of the association between APP and other variables in the multivariable analysis model.

### Ethics approval and consent to participate

Ethical approval was received from the ethical review committee of the School of Pharmacy of the University of Gondar. This was granted with SOPS/206/2014. All study participants were informed about the purpose of the study and gave their written consent to participate in this study. Their unwillingness to participate could not have affected the service they received. Participants’ privacy was guaranteed, and personal identifiers were not used. All the data were sufficiently synonymized. The study was conducted based on the Declaration of Helsinki.

## Results

### Sociodemographic characteristics of patients

From a total of 430 approached samples, 422 (with a response rate of 98.1%) eligible patients with schizophrenia were included in the study. The majority (58.3%) were females, with a mean (±SD) age of 36.4 (±11.5) years. More than half (51.2%) of participants were married, and more than two-thirds (69.4%) lived in urban areas. More than a quarter (28.7%) of the participants’ educational level was high school, and around one-fifth (22.5%) of them were privately employed. More than three-quarters (76.1%) of participants had a monthly income above 1200 Ethiopian birr (**Table 1**).

**Table 1:** Sociodemographic characteristics of outpatient schizophrenia patients (N = 422).

Variables	Categories	Frequency	Percentage	Mean (±SD)
Sex	Male	176	41.7	
	Female	246	58.3	
Age	18–24	57	13.5	36.4 (±11.5)
	25–34	139	32.9	
	35–44	129	30.6	
	≥45	97	23	
Marital status	Single	160	37.9	
	Married	216	51.2	
	Divorced	37	8.8	
	Widowed	9	2.1	
Residence	Urban	293	69.4	
	Rural	129	30.6	
Religion	Orthodox	317	75.1	
	Muslim	94	22.3	
	Protestant	8	1.9	
	Catholic	3	0.7	
Educational level	No formal education	127	30.1	
	Primary (1–8 grades)	70	16.6	
	Secondary (9–12 grade)	121	28.7	
	Diploma and above	104	24.6	
Occupation	Farmer	79	18.5	
	Government employee	68	16.1	
	Private employee	95	22.5	
	Daily labor	14	3.3	
	Student	44	10.4	
	Jobless	56	13.3	
	Housewife	66	15.6	
Monthly income in birr	<750 birr	90	21.3	
	750–1199 birr	11	2.6	
	≥1200 birr	321	76.1	

### Clinical and substance related characteristics of participants

In terms of clinical characteristics, nearly half of the participants (46.9%) and nearly three-quarters (73.2%) had been ill for less than 5 years. One-seventh (14.5%) of patients had other comorbid illnesses, including hypertension (14, 3.3%) and diabetes mellitus (13, 3.1%). One-quarter of respondents (25.6%) had two or more inpatient admissions, and 32.2% were substance users. More than two-fifths (42.6%) of those who used psychoactive substances used alcohol (**Table 2**).

**Table 2:** Clinical and substance use related variables of outpatient schizophrenia patients (N = 422).

Variables	Categories	Frequency	Percent
Duration of illness (in year)	< 5	198	46.9
	5–10	146	34.6
	>10	78	18.5
Duration of treatment (in year)	<5	309	73.2
	5–10	73	17.3
	>10	40	9.5
Presence comorbidity	Yes	61	14.5
	No	361	85.5
Type of comorbidity	Hypertension	14	3.3
	Diabetes	13	3.1
	Gastritis	12	2.8
	Epilepsy	6	1.4
	HIV	5	1.2
	Respiratory disorder	5	1.2
	Cardiac disorder	4	0.9
	Hepatitis	2	0.5
Number of admissions	None	182	43.1
	One	132	31.3
	≥two	108	25.6
Substance use	Yes	136	32.2
	No	286	67.8
Type of substance use	Alcohol	58	42.6
	Cigarette	23	16.9
	Khat	48	35.3
	Others*	7	5.2

Others*, Shisha, DM: Diabetes Mellitus, HIV: Human Immunodeficiency Virus

### Prevalence of APP and medication-related characteristics

In this study, the prevalence of APP was 22.7% (95% CI: 19.0, 27.0), and among those on polypharmacy, more than three-fourths (80.2%) of the participants used combined FGA. About one-fourth (24.4%) of patients took haloperidol and 13.5% of respondents were taking a combination of haloperidol and fluphenazine decanoate. In patients taking adjuvant medication, about one-third (34.5%) took amitriptyline. Nearly half (48.4%) of participants took the SGA (**Table 3**).

**Table 3:** Medication related characteristics of outpatient schizophrenia patients (N = 422).

Variables	Categories	Frequency	Percent
Type of antipsychotics	FGA	122	28.9
	SGA	204	48.4
	FGA + SGA	19	4.5
	FGA + FGA	77	18.2
Antipsychotic medication recorded	Haloperidol	103	24.4
	Chlorpromazine	19	4.5
	Risperidone	179	42.4
	Olanzapine	25	5.9
	Haloperidol + Fluphenazine decanoate	57	13.5
	Chlorpromazine + Fluphenazine decanoate	20	4.7
	Risperidone + fluphenazine deconate	15	3.6
	*Others	4	1.00
Adjuvant medications	Amitriptyline	39	34.5
	Fluoxetine	21	18.7
	Trihexyphenidyl	18	15.9
	Diazepam	17	15.0
	Sodium valproate	12	10.6
	**Others	6	5.3
Antipsychotic polypharmacy	Yes	96	22.7 (95% CI: 19.0, 27.0)
	No	326	77.3
	FGA + SGA	19	19.8
	FGA + FGA	77	80.2

*Others Risperidone + Haloperidol, Olanzapine + Fluphenazine decoante

**Others: Phenobarbital, Carbamazepine, Lorazepam

FGA, First Generation Antipsychotics; SGA, Second Generation Antipsychotics

### Factors associated with antipsychotic polypharmacy

On multivariate analysis, duration of illness, duration of treatment, number of admissions, and substance use were significantly associated with APP. Consequently, patients who had five to ten years of illness were 2.88 times more likely to be on APP than those who had a duration of illness of less than five years (AOR = 2.88, 95% CI: 1.49; 5.59). Regarding the duration of treatment, patients who were on treatment for more than ten years were 3.79 times more likely to be on APP compared with those who were on treatment for less than five years (AOR = 3.79; 95% CI: 1.05, 13.62). Similarly, patients who were admitted once and twice or more were 2.39 and 4.93 times more likely to be on APP than those who had no previous admission history, (AOR = 2.39; 95% CI: 1.20, 4.77) and (AOR = 4.93; 95% CI: 2.52, 9.64), respectively. Concerning substance use, respondents who used psychoactive substances were 2.58 times more likely to be on receive APP than those who were not substance users (AOR = 2.58; 95% CI: 1.49, 4.47) (**Table 4**).

**Table 4:** Univariable and multivariable binary logistic analysis for antipsychotic polypharmacy and associated factors of outpatient schizophrenia patients (N = 422).

Variables	Categories	APP		95% CI		P -value
		Yes	No	COR	AOR	
Marital status	Unmarried	52	154	1.32 (0.83, 2.08)	1.12 (0.60, 2.06)	0.715
	Married	44	172	1.00	1.00	
Residence	Urban	73	220	1.52 (0.90,2.58)	1.33 (0.53, 3.30)	0.540
	Rural	23	106	1.00	1.00	
Occupation	Farmer	14	65	0.87 (0.38,2.02)	0.74 (0.23, 2.34)	0.616
	Gov`t employee	14	54	1.05 (0.45,2.46)	0.75 (0.26, 2.09)	0.585
	Private employee	24	71	1.37 (0.64,2.95)	0.63 (0.24, 1.66)	0.360
	Daily labor	6	8	3.05 (0.90,10.35)	2.74 (0.68, 11.04)	0.155
	Student	8	36	0.90 (0.34,2.40)	1.08 (0.32, 3.57)	0.896
	Jobless	17	39	1.77 (0.77,4.08)	0.84 (0.29, 2.41)	0.749
	Housewife	13	53	1.00	1.00	
Duration of illness	>10 year	30	48	4.75 (2.53, 8.93) *	2.28 (0.69, 7.50)	0.172
	5–10 years	43	103	3.17 (1.81, 5.57) *	2.88 (1.49, 5.59) *	**0.002**
	< 5 year	23	175	1.00	1.00	
Duration of treatment	>10 year	23	17	5.85 (2.94, 11.66) *	3.79 (1.05, 13.62) *	**0.040**
	5–10 year	15	58	1.11 (0.59, 2.11)	0.66 (0.28, 1.55)	0.343
	< 5 year	58	251	1.00	1.00	
Presence of comorbidity	Yes	18	43	1.51 (0.83, 2.78)	0.97 (0.49, 1.93)	0.951
	No	78	283	1.00	1.00	
Number of previous admissions	≥ Twice	48	60	7.28 (3.93, 13.51) *	4.93 (2.52, 9.64) *	**< 0.001**
	once	30	102	2.68 (1.42,5.05) *	2.39 (1.20, 4.77)	**0.013**
	None	18	164	1.00	1.00	
Substance use	Yes	50	86	3.03 (1.89, 4.85) *	2.58 (1.49, 4.47) *	**0.001**
	No	46	240	1.00	1.00	

* P < 0.05, AOR: Adjusted Odds Ratio, APP: Antipsychotic Polypharmacy, CI: Confidence Interval, COR: Crude Odds Ratio, **bold figures**; statistically significant variables

## Discussion

Even after repeated exhortations and recommendations to avoid APP and the absence of a compelling pharmacological rationale, co-prescribing antipsychotics continues to remain a common and widely used practice. The likelihood of non-compliance related to a more complicated regimen, an increased adverse effect burden mediated by drug-drug interactions, and exposure to high-dose antipsychotic medication are all issues correlated with the prescription of combined antipsychotics [11]. Aiming at assessing APP and associated factors in patients with schizophrenia, the current study found that the overall prevalence of APP was 22.7% (95% CI: 19–27). Duration of illness (AOR = 2.88; CI: 1.49, 5.59), duration of treatment (AOR = 3.79; CI: 1.05, 13.62), number of admissions (AOR = 4.93; CI: 2.52, 9.64), and substance use (AOR = 2.58; CI: 1.49, 4.47), were significantly associated with APP.

In this study, the prevalence of APP was 22.7% (95% CI: 19–27), which is comparable with studies in Germany (20%) [12], Hong Kong (24.2%) [13], and Korea (20.4%) [14]. However, this finding is lower than that of the study done at Amanuel Mental Specialized Hospital, Ethiopia (28.2%) [2]. A possible explanation might be a difference in clinical settings, such as refractoriness to medical treatment and referral centers. The study conducted in Nigeria indicated that the magnitude was 50.9%, which was very high compared to this study [15]. In addition, the magnitude of APP was reported in studies conducted in Australia (39%) [16], China (45.7%) [17], and Poland (47.3%) [18], which were higher than in the current study. On the contrary, this finding is higher than a nationwide report in the USA (12%) [19], in North Carolina (13%) [20], and in Spain (13.8%) [21]. The probable explanation for inconsistencies in the magnitude of APP among different studies might be due to differences in the sociodemographic characteristics of the study participants. This might be due to differences in inclusion and exclusion criteria, sample size, and clinical setup. More than that, some studies have used diverse inclusion and exclusion criteria, like a cut point to consider as AAP. This ranges from two or more antipsychotics given at a time [19] up to 12 weeks and above [21]. The findings may implicate physicians could be vigilant to minimize prescribing a combination of medications as much as possible. Patients also APP need a strict follow-up to minimize possible adverse effects from combined antipsychotics.

About 77% of those taking APP were also taking conventional antipsychotics. This finding is consistent with research conducted in Addis Ababa, Ethiopia [2], and Nigeria [22]. This could be because they are less expensive and more widely available than atypical antipsychotics. Clinicians prefer first-generation antipsychotics for these reasons. The finding may suggest that physicians could be involved in identification of other possible causes of treatment failure before addition of another antipsychotic.

In terms of illness duration, patients with illnesses lasting more than 5 years were nearly three times more likely to be on APP compared with patients with illnesses lasting less than 5 years. This finding is consistent with research from Finland [23] and Singapore [24]. Long periods of illness may be associated with refractory responses to antipsychotic treatment, which clinical practitioners attempt to overcome by combining antipsychotics. The current study also revealed that patients with a longer treatment duration were more likely to receive APP compared with patients with a shorter treatment duration. This is consistent with previous research from Ethiopia [2] and Malaysia [25]. The possible explanation is that after starting treatment, these patients may have interrupted their follow-up after achieving remission, and they may have had a poor outcome over time. Patients with poor treatment outcomes may be placed on a combined antipsychotic treatment regimen. Healthcare providers need to follow and minimize the use of unnecessary APP for patients with longer durations of illness and treatment.

This study also showed that patients with multiple admissions were more likely to be on the APP. This finding is consistent with research from Ethiopia [2], South Africa [26], and India [27]. The most likely explanation is that hospitalization is associated with increased disease severity, resistance to medical therapy, noncompliance with standard regimens, and drug-related side effects. All of the aforementioned issues may aggravate schizophrenia symptoms, predisposing patients to frequent hospitalization and leading to APP rather than monotherapy.

In the current study, patients who used psychoactive substances were twice as likely to be on APP. This finding is consistent with research from Ethiopia [2], South Africa [15], and Nigeria [26]. This could be because a poor response rate for positive psychotic symptoms and non-adherence to antipsychotics are positively correlated with substance use, which could lead to antipsychotic combining. Patients who use psychoactive substances require psychological support to withdraw from psychoactive substances and adhere to their medications.

In general, the current study highlighted the magnitude of APP and its associated factors in schizophrenia patients. This study presented comprehensive findings using a multicenter and relatively large study sample than previous findings. This could be helpful to draw conclusions from and used as a baseline and source for future studies in the area. The findings could indicate that a high level of APP necessitates an intervention to reduce the APP-related burden. Patients could thus be closely monitored. Modifiable independent determinant factors need to be modified to the greatest extent possible.

### Strength and limitation of study

Despite the fact that this study is multicenter with a relatively large sample size, which is preferable for generalizability, due to the inherent nature of a cross-sectional study, assessing a causal relationship between the independent and dependent variables was not possible. As a result, future research would be welcomed using a prospective follow-up study. Clinical data relating to disease severity were difficult to assess. Substance use assessment is prone to social desirability bias.

## Conclusion and recommendation

This study revealed that APP was recorded in a considerable number of patients. Patients with a longer duration of illness and treatment, frequent admissions,, and those who use substances need close follow-up and attention. Clinicians should use standard prescribing guidelines to improve antipsychotic use. Besides, healthcare professionals working in hospitals should screen for substance use regularly and provide counseling on psychoactive substances cessation. Future researchers could examine the causal association between APP and potential predictor variables.

## Supporting information

S1 File Data set used in generating and analyzing the finding. (XLSX)

## List of abbreviations

APM: Antipsychotic Monotherapy
APP: Antipsychotic Polypharmacy
ASSIST: Alcohol Smoking and Substance Involvement Screening Test
EPS: Extrapyramidal Side Effect
FGA: First Generation Antipsychotic
FHCSH: Felege-Hiwot Comprehensive Specialized Hospital
SGA: Second Generation Antipsychotics
TGCSH: Tibebe-Ghion Comprehensive Specialized Hospital
UOGCSH: University of Gondar Comprehensive Specialized Hospital
